# A feasibility and pilot study of a “lifelong learning” intervention for people with dementia

**DOI:** 10.1186/s40814-024-01493-5

**Published:** 2024-05-01

**Authors:** Ann Lykkegaard Soerensen, Diana Schack Thoft, Alison Ward, Jackie Campbell

**Affiliations:** 1 University College of Northern Denmark, Aalborg, Denmark; 2 University of Northampton, Northampton, UK

**Keywords:** Dementia, Cognitive stimulation, Lifelong learning, Feasibility

## Abstract

**Background:**

Developing evidence for the use of psychosocial interventions for people with dementia is a research priority. This pilot study aimed to provide variability estimates for a set of outcome measures that would inform the development of a more extensive controlled study. The larger study will seek to explore the effect of attending a lifelong learning intervention for people with dementia compared to receiving treatment as usual. This pilot and feasibility stage also analysed how data collectors and researchers evaluated the use of the outcome measures in a sample of people with mild to moderate dementia.

**Methods:**

Before initiating the pilot study, a participant consultation was conducted with people with dementia, who attend a lifelong learning service known as a dementia school, and their teachers. From this consultation, the research outcomes identified were the mini-mental state examination (MMSE), Quality-of-Life Alzheimer’s Disease (QoL-AD), General Self-Efficacy Scale (GSE), Rosenberg self-esteem scale, and the Friendship scale. The following study was divided into two steps. In step 1, participants were people with dementia attending a dementia school (intervention group) or usual services (control group). The participants were tested at baseline and at a 6-month follow-up. Data were collected between November 2018 and July 2019. In step 2, feasibility and acceptability issues with the recruitment of participants, data collection process, and outcome measures, identified in step 1, were evaluated through a data collector focus group.

**Results:**

Fifty-five people with dementia were included in the analysis. Step 1 provided estimates of changes from baseline to follow-up, and ancillary standard deviations were supplied for all outcome measures. Step 2 provided reflections on the feasibility and acceptability of the intervention, data collection, and outcome measures. This included views on how people with dementia experience participating in a test situation.

**Conclusions:**

This study provided estimates of change and variability in the outcome measures. Additionally, issues regarding data collection were identified and should be addressed in future studies. The project demonstrated how to support people with dementia to participate in research that is meaningful to them.

**Trial registration:**

According to national legislation, registration with a database of clinical studies was optional, as the study evaluated existing activities rather than a clinical intervention.

**Supplementary Information:**

The online version contains supplementary material available at 10.1186/s40814-024-01493-5.

## Key messages regarding the feasibility

• What uncertainties existed regarding the feasibility? To inform the sample size for a more extensive randomised study of the lifelong learning service, this feasibility project aimed to ascertain the following: (a) the participation of people with dementia in selecting outcome measures that held personal significance for them; (b) the estimation of variability in a chosen set of outcome measures applied to a sample of people with dementia; (c) the recruitment feasibility of participants meeting the inclusion criteria, and their ability to complete the designated measures; and (d) the data collectors’ evaluation of the burden on people with dementia during the test administration and data collecting process.

• What are the critical feasibility findings? Individuals with mild to moderate dementia actively engaged in research on the lifelong learning service by selecting and completing outcome measures that held personal significance for them and their involvement in the service. To facilitate the participation of research sites, additional resources may be required to support staff in assessing participant eligibility and documenting demographic data. The number of participants included in the final analysis was 30 from the lifelong learning intervention group and 25 from the control group. Insights from the data collector focus group presented various experiences using the different outcome measures. These included recognising the importance of using pen and paper rather than a personal computer (PC) or tablet when collecting data and the value of allowing time for meaningful conversations between conducting the measures. While all data collectors found the outcome measures feasible, they also suggested other or additional outcome measures that warrant consideration in future studies.

• What are the implications of the feasibility findings for the design of the main study? The feasibility findings of the pilot study suggest that a more extensive randomised study is viable. This larger study would investigate the impact of the lifelong learning intervention compared to usual services for people with dementia. Furthermore, power and sample size calculations provide insights into the variability of the five outcome measures employed in this preliminary phase. The results emphasise the importance of involving people with dementia in defining the essential outcomes of psychosocial intervention in future studies. However, a cautious note is raised to closely monitor the research’s direct impact on participants. The findings will inform a fully powered randomised study of the lifelong learning service.

## Background

Being diagnosed with dementia has severe and far-reaching consequences physically, emotionally, and psychologically for patients, family, and carers, and thus, effective interventions to relieve these consequences are required [1, 2]. Psychosocial interventions are becoming an integral part of the treatment of people with dementia in Europe and globally, where the focus is on supporting those living with dementia, delaying its progression [3], and providing evidence-based non-pharmacological interventions [4].

Ward et al. suggest that an innovative lifelong learning approach to provide “education in the classroom” tailored for people with dementia can stimulate cognition and memory [5]. In this context, researchers emphasise the underlying philosophy of “lifelong learning” and its potential benefits for empowerment and dignity in later life [6]. Lifelong learning means continually learning and developing, remaining socially engaged, and maintaining a life offering the experience of joy [7]. In Denmark, there is a strong tradition for lifelong learning which goes back to N. F. S. Grundtvig (1783–1872), a theologist who, in the aftermath of the time of enlightenment, brought out the idea of providing all people free access to cultural and scientific knowledge [8, 9]. Based on that historical background, a dementia school in northern Denmark offers education for people with dementia in a school environment. Lessons are specially developed to support cognitive function, quality of life, problem-solving, self-esteem, and social engagement, and this model has been inspired by cognitive training (CT) and cognitive stimulation therapy (CST) [10]. Qualitative research of the school participants’ experiences with the service supports the lesson aims as participants emphasise self-perceived impacts in these domains [11–14]. There is growing evidence about the effect and impact of psychosocial interventions, which has also emphasised the need to distinguish between the hallmarks of the various approaches. Examples of such techniques are cognitive stimulation (CS), cognitive stimulation therapy (CST), and cognitive training (CT) [15]. CS is a psychological intervention that enhances cognition through targeting cognitive and social functioning [16]. For example, CS incorporates reality orientation, attention, memory, language, and problem-solving [3]. A variant of CS is CST, which is an evidence-based programme that was developed following a Cochrane review and a systematic review of the use of reality orientation therapy for people with dementia [10, 17, 18]. CST consists of 14 × 45-min group sessions with a manual to guide the sessions [19]. In contrast to CST, CT is often defined as “guided practice on a set of standard tasks designed to reflect cognitive functions, for example, memory or executive function” [20].

Research suggests the benefits of CS can be wide reaching, affecting cognition and socialisation, including communication and quality of life [21, 22]. However, the number of high-quality randomised controlled trials still needs to be higher [23]. Comparisons between studies using CS may also be difficult due to heterogeneity in the study design, including a lack of consistency in using validated measures, sample sizes, and use of comparison groups [14].

Much evidence on the effectiveness of interventions aimed at dementia is focused on reducing the burden of symptoms characterising the condition rather than what is experienced as crucial by those living with dementia [24]. Research is now embracing the patients and their carers in determining important outcomes in dementia research [25]. The emphasis on involving patients, and in some cases, their carers, in randomised controlled studies of good quality will require valid and reliable estimates of effects and variability in outcome measures and evaluating the feasibility of undertaking such trials.

### Aim

This pilot study sought to provide estimates of variability for a set of outcome measures considered for a more extensive controlled study of the effect of attending a lifelong learning (LL) intervention for people with dementia compared to receiving treatment as usual. The pilot study also investigated how data collectors and researchers experienced the feasibility of using the outcome measures in a population of people with mild to moderate dementia.

## Method

### Setting and study design

The dementia school operates as a day programme. The school represents a distinctive model that combines a community-run organisation with a school facility and offers tailored cognitive classes. Participants’ engagement is solely focused on the cognitive and educational experience provided within the school. There are no other similar health-related services; this is noteworthy as the dementia school operates independently, offering a standalone educational experience. This setting operates on the principle of community engagement, drawing on the expertise of teachers familiar with the challenges of dementia.

**Fig. 1:**
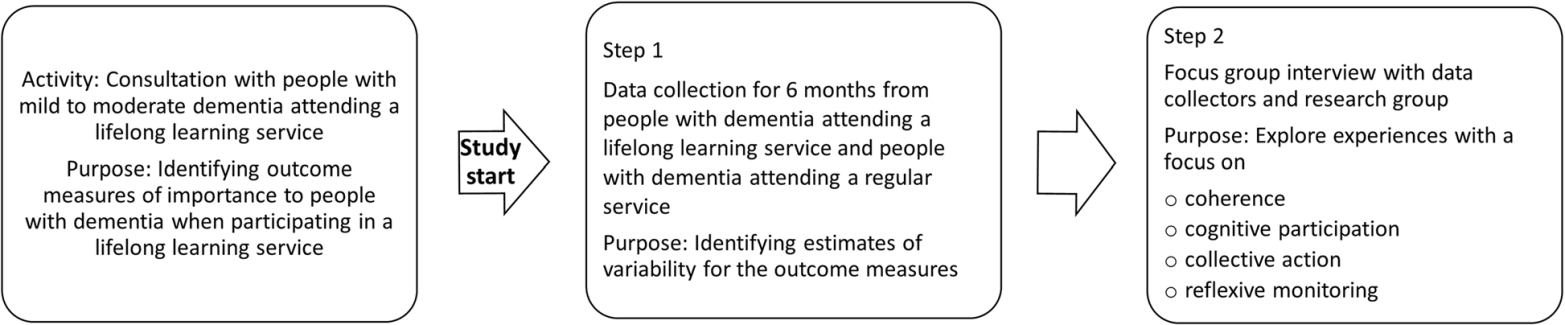
Overview of the study

Before undertaking the pilot study, a participant consultation involving students and teachers from the dementia school was conducted. The pilot study fell in two steps. In step 1, the pilot study participants were people with dementia who attended the lifelong learning dementia school (intervention) or a dementia day service (control). These services are not time limited but are offered for as long as the person with dementia experiences it as meaningful and staff consider it beneficial. Therefore, the length of attendance varied between participants. The activities for both intervention and control groups were in a similar timeframe. Individuals in the control and intervention groups were assessed at the beginning and end of 6 months with cognition, quality of life, self-efficacy, self-esteem, and socialisation measures. Thus, there needs to be a clear baseline as participants were already engaged in the different services. However, the pilot study does not seek to estimate any effect sizes — only the variability of outcome measures. In step 2 of the pilot study, feasibility and acceptability issues with recruiting people with mild to moderate dementia, data collection process, and outcome measures were evaluated in a focus group interview and analysed qualitatively. Participants in the focus group interview were data collectors and the research team. An overview of the study phases is shown in Fig. 1. This pilot study was conducted using the CONSORT Extension to Pilot and Feasibility Trials checklist, where possible. The study could not strictly adhere to RCT guidelines as it was a non-randomised controlled study with a pragmatic approach.

#### Participant consultation

The participant consultation conducted before the pilot study included interviews with two teachers and focus groups with 23 people with dementia attending a dementia school (9 women and 14 men). Teachers were actively involved in the consultation process due to their intimate knowledge of participants, allowing them to discern subtle signs of fatigue or discomfort. Their presence was considered crucial for gaining a comprehensive understanding of individual needs, thereby enhancing the reliability of the consultation. This decision aimed to uphold ethical considerations and prioritise participants’ comfort and genuine engagement. The teachers were informed that their role was to support the voice of the participants, for example, by supporting them in writing down suggestions. The consultation explored how people with dementia experienced the lifelong learning programme and asked what would be essential to research for those receiving and delivering the programme. Factors deemed meaningful included socialising with peers, well-being, learning and the learning environment, and insight into one’s life situation. In a separate consultation, the teachers identified the following areas: to investigate the assessment of cognition and memory, quality of life, verbal fluency, stress, and well-being. From these findings and two previous qualitative studies conducted with this service, the research team identified the measures to be included in the study [11, 12]. This ensured that the outcome measures were essential experiences for those attending the lifelong learning programme and those who deliver it.

#### Step 1 of the pilot study

##### Participants

Recruitment for the intervention group took place in four dementia schools. Participants in the control group were recruited from six different dementia services across five municipalities. Before recruitment, the researchers conducted an introductory meeting with staff at each service to ensure consistency. Recruitment was conducted between November 2018 and February 2019, where participants were assessed at inclusion and again after 6 months, from April 2019 to July 2019. Participants were recruited if they (1) had a diagnosis of dementia, (2) attended a service providing the lifelong learning programme or another dementia service, and (3) could provide informed consent. Participants were excluded if they attended fewer than 10 times during the data collection. Allocation was determined by service offered in the individual/included municipalities.

##### Teachers and data collection

The staff at the dementia school are referred to as “teachers”. They had varied professional backgrounds, but all possessed health or education expertise. Staff at the facilities providing the usual services for people with dementia had health backgrounds.

In step 1 of the pilot study, tests were conducted by an associate professor (PhD) with experience in dementia and nursing, as well as two assistant professors in nursing. One of the assistant professors was also a member of the research group.

The data collectors were trained for consistency in the assessment approach. The data collectors were not blinded as they attended different services. All tests were conducted according to an agreed protocol and in the same order. The team met twice to review the process and ensure the tests were consistently administered.

##### Ethics

The Ethics Committee of Northern Denmark confirmed compliance with national rules for health science research projects. The study adhered to the General Data Protection and Privacy Regulations (GDPR) within the research teams’ institution. Registration with a database of clinical studies was not required as the study was evaluating existing activities and not a clinical intervention. Participants were provided with dementia-friendly information and asked to provide informed written consent. Navigating the ethical landscape of research involving individuals with dementia is complex due to their fluctuating ability to provide informed consent. Balancing respect for autonomy with the need for protection requires careful consideration and adaptable approaches to ensure the well-being and participation of individuals throughout the research process. The recruitment process was based on collaborating with the participant, their relatives, and service staff. This process aimed to allow participants to engage in the research, understand what taking part would mean, and provide informed consent to participate.

No harm was observed to the participants in either the intervention or control groups.

### Intervention

#### Lifelong learning (LL) group

These were people with dementia receiving a lifelong learning intervention. Participants attend 1–2 days a week and take part in cognitive classes for 3–4 hours a day, depending on the progression of their dementia. Details of the classes and the aims of the dementia school can be found in previously published papers [5, 11, 12]. This programme is designed for people with early-stage dementia to provide ongoing education to support a person with dementia’s cognitive function, decision-making, and activities of daily living, supporting them to retain their independence for as long as possible. The core focus of the intervention is on the delivery of cognitive stimulation, with a supplementary curriculum that changes according to the interests of those attending [5, 11, 12, 26]. The intervention has been designed into three tiers to support changes and declines in dementia. The intervention sites work closely with their local dementia coordinators and nurses to assess people’s stage of dementia and ensure they are in the most appropriate tier for their level of functioning and to provide a service for people to move onto when the intervention is mutually agreed to no longer be of interest or benefit. Since 2000, the intervention has been delivered by qualified teachers and has been based on theories derived from cognitive stimulation therapy [10], neuropsychology [27], and education [28].

#### Control group

The control group engaged in activities delivered via dementia services at day-care centres or other social places, including dementia cafés, offering social and physical activities with instructors. Activities included crafts, reading newspapers, and seasonal activities such as making Christmas and Easter decorations. People with dementia attended 1–3 days a week for 3–5 hours.

### Outcome measurements

The outcome measures chosen were based on the consultation process results, qualitative literature in this field, and a narrative literature review of existing research on CS, CST, CT, and rehabilitation [14]. A review of these sources identified the following domains: cognition, friendship, quality of life, self-esteem, and self-efficacy. The accessibility of validated tests in Danish and the level of expertise required to administer the measures were considered when selecting appropriate measures. Following an extensive review of the measures available, five tests were chosen. To avoid overburdening the participants, the research team aimed to limit the amount of time for each assessment to a maximum of 1 h. The following measures were used:

1. Mini-mental state examination (MMSE) [29]: The MMSE is used to support the diagnosis of dementia and to assess cognitive decline in patients with dementia. It consists of questions to test mental ability, memory, language, and attention. This is a well-established, validated measure used in dementia research to assess changes in cognition over time. The decline in cognition may be a consequence of dementia. However, an increased or stabilised score on the MMSE could indicate an intervention response [30].
2. Quality-of-Life Alzheimer’s Disease (QoL-AD) [31, 32]: This is a 13-item measure using a 4-point Likert scale and is a patient-self-administered and caregiver-administered measure. Only the patient version was used in this study. The measure aims to provide psychometric data on the perceived quality of life in patients with Alzheimer’s disease.
3. General Self-Efficacy Scale (GSE) [33]: This is a 10-item scale to measure self-efficacy, i.e. an individual’s belief in their capacity to behave or act in a certain way [34], which relates to emotion, optimism, and work satisfaction. Items are answered on a 4-point Likert scale. Previous research has shown that self-efficacy for those diagnosed with dementia is associated with greater control over life situations, improved confidence, and a more positive sense of control [35].
4. Rosenberg Self-Esteem Scale [36]: This is a 10-item scale to measure global individual self-esteem, including positive and negative feelings of the self. Items are answered on a 4-point Likert scale. Self-esteem has been associated with happiness and improved relationships [37]. For people with dementia, a diagnosis can have a negative impact on an individual’s self-esteem. It can indirectly impact their health and how they manage their diagnosis [38].
5. Friendship scale [39]: This is a 6-item measure using a 5-point Likert scale. The measure was developed to explore issues of isolation in older adults. Friendship was an important theme emerging from the work of Thoft [11] and Ward [5] about their experiences of being a student at the Dementia School in Northern Denmark.
6. Depending on personal preferences, tests were conducted face to face in a quiet room at each service or at the individual’s home. All the participants were able to complete all tests. Time was provided for breaks if needed, and participants were provided with refreshments.

### Data analysis

Statistical analysis was carried out using IBM SPSS version 26. Descriptive statistics were utilised to present demographic characteristics. Estimates of change and variability were given as mean and standard deviation for each outcome measure for the LL and control groups. Per protocol, analysis was carried out, and only cases with complete data were included. Although it is acknowledged that this affects the power of the study, it was felt essential to reflect the actual data collected in this pilot study rather than imputing missing data or using a last observation carried forward approach.

#### Step 2 of the pilot study

##### Participants

The interview group comprised three individuals, with one member serving as both a data collector and a research team member. However, there was no further overlap between the two groups.

##### Feasibility

The feasibility evaluation encompassed an analysis conducted through a focus group interview involving data collectors and the research team. The primary focus was on the acceptability and feasibility of outcome measures, spanning participants, data collectors, and the administering research team. Key areas assessed included recruitment processes, the acceptability of data collection procedures, and the relevance of outcome measures. Methodological considerations, such as data completeness and the appropriateness of outcome measure choices, were also addressed. Notably, the feasibility and acceptability assessment followed an open and inductive approach, avoiding predetermined thresholds. This exploratory and responsive methodology aimed at capturing nuanced insights and emergent factors that could impact outcomes. This methodological choice resonates with the pragmatic and exploratory nature of the study.

##### Data analysis

One interviewer conducted the focus group interview and is also the manuscript’s first author (A. L. S.). The interviews were recorded in Microsoft Teams. The applied analysis technique was deductive, as the initial codes driven by the project aims aligned with the interview. The focus group was structured according to a discussion guide informed by normalisation process theory (NPT) [40]. NPT’s core constructs are coherence (how people “make sense” of the challenges they face), cognitive participation (the relational work people are willing to invest in the intervention to make the intervention work), collective action (how people operationalise the intervention and which resources are needed to make the intervention work), and reflexive monitoring (how people appraise and understand the intervention) [40]. See Appendix 1 for the discussion guide applied in the focus group interviews. A thematic approach based on Braun and Clarke [41] [42] was used to conduct the analysis. Using their six-stage approach, the data was read and coded in line with the identified codes. These initial codes were presented to and discussed with the rest of the research group, and the group’s discussion steered the formation of themes.

## Results

### Step 1 of the pilot study

#### Participant demographics

**Fig. 2:**
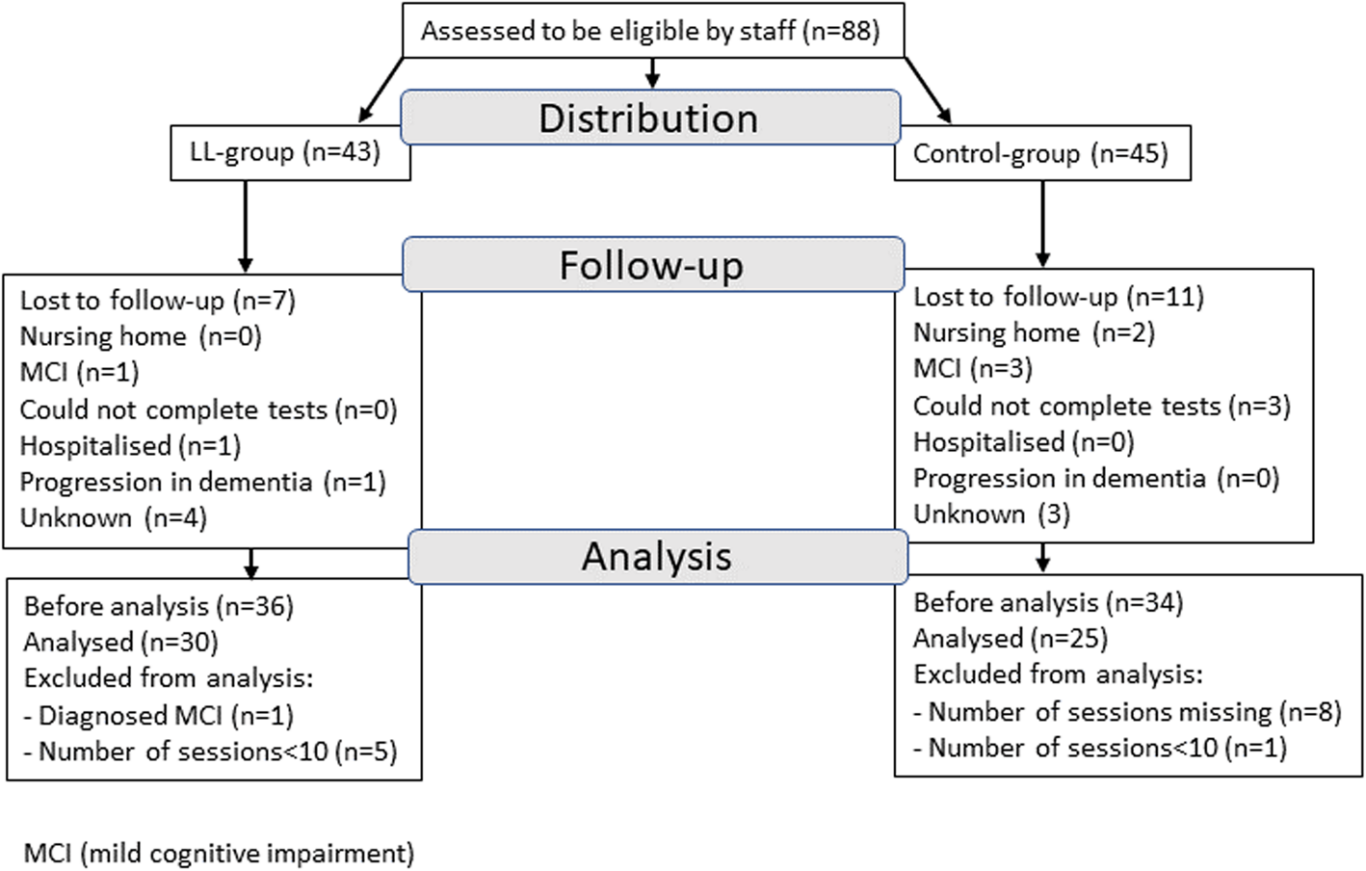
Participant flowchart

Staff from the control and intervention services were recruited through a convenience sample. The staff recruited 88 participants (43 in the LL group and 45 in the control group). Following attrition and exclusions, the final number of participants was 55, 30 in the LL group, and 25 in the control group (see flowchart in Fig. 2 for details).

There were statistically significant differences between the groups for age, where the LL group was younger on average (median 72.5 years compared with 76.0 years for the controls) and for MMSE score, where the LL group had a higher score on average (LL group had a mean of 21.83 compared with 18.44 for the control group). Participant demographics can be seen in Table 1. The analysis did not show other significant differences in participant demographics.

**Table 1:** Baseline data

Characteristics	Control group	LL group
	*n* = 25	*n* = 30
Age in years (median, IQR)	76.0 (72.5–82.5)	72.5 (62.8–74.5)
Gender, *n* (%)		
Male	16 (45.7)	19 (54.3)
Female	9 (45.0)	11 (55.0)
Dementia type, *n* (%) (two missing in the control group)		
Alzheimer’s disease	17 (47.2)	19 (52.8)
^a^Other types of dementia	6 (35.3)	11 (64.7)
Level of education, *n* (%) (one missing in the control group and one missing in the LL group)		
Elementary	12 (57.1)	9 (42.9)
Skilled work/short education	11 (47.8)	12 (52.2)
Shorter college/university degrees	1 (11.1)	8 (88.9)
Long university degrees	0 (0)	0 (0)
Participates in other activities, *n* (%) (five missing in control group)		
No	8 (30.8)	18 (69.2)
Yes	12 (50)	12 (50)
Housing, *n* (%)		
Care home or other facility	0 (0)	0 (0)
Own home	25 (100)	30 (100)
Other	0	0 (0)
Number of sessions (median, IQR)	25 (17–35.5)	22 (15–25)
Baseline for outcome measures		
MMSE mean (SD)	18.44 (5.17)	21.83 (3.44)
QOL-AD mean (SD)	37.24 (5.52)	37.7 (4.04)
GSE mean (SD)	30.46 (6.26)	28.81 (7.10)
Self-esteem scale mean (SD)	19.05 (5.75)	20.74 (4.84)
Friendship scale mean (SD)	20.02 (3.90)	20.22 (3.23)

^a^Due to limitations in accessing detailed register data, specific information on dementia subtypes was not available for individual participants. The category “Other types of dementia” encompasses various subtypes beyond this study’s detailed differentiation scope

The mean changes and standard deviation for the outcome measures can be seen in Table 2.

**Table 2:** Changes in outcome measures (mean, SD) for the LL group and control group

	Control group (*n* = 25)			LL group (*n* = 30)		
	Baseline	Follow-up	Change (SD)	Baseline	Follow-up	Change (SD)
MMSE mean (SD)	18.44 (5.17)	18.52 (5.64)	0.08 (2.84)	21.83 (3.44)	22.13 (4.23)	0.30 (4.19)
QOL-AD mean (SD)	37.24 (5.52)	37.08 (6.30)	−0.16 (5.01)	37.7 (4.04)	37.33 (5.13)	−0.37 (3.57)
GSE mean (SD)	30.46 (6.26)	29.56 (6.18)	−0.90 (4.71)	28.81 (7.10)	28.96 (5.31)	0.16 (4.62)
Self-esteem scale mean (SD)	19.05 (5.75)	19.72 (5.37)	0.67 (3.62)	20.74 (4.84)	19.97 (5.74)	−0.77 (4.49)
Friendship scale mean (SD)	20.02 (3.90)	19.36 (4.60)	−0.66 (3.65)	20.22 (3.23)	20.97 (3.21)	0.74 (2.35)

#### Outcome measures

The estimates of mean and standard deviations of the outcome measures provided in Table 2 can inform sample size calculations for future efficacy studies.

Step 2 of the pilot study

#### Feasibility and acceptability of the intervention

##### Recruitment of people with dementia

The number of potential participants varied across the LL and dementia services due to the progressive nature of dementia and the different ways the services are organised (with varying attendance). Recruitment was undertaken by staff at each service, and challenges were experienced about participant recruitment. When data collectors (DCs) arrived at the sites, there were instances where a participant did not fulfil the criteria for participation (for example they had a diagnosis of MCI). Entry criteria had been discussed at the introduction meetings; however, additional training on recruitment could have mitigated this. Contacting the services for follow-up interviews on the recruitment process has not been possible. The following quote is from the NPT focus group:

I don’t think the resources were there (at the sites), but with lots of time and resources, a better structure and recruitment could have been established... so recruitment forms could have been filled out correctly from the beginning. (DC2)

This suggests that future studies could benefit from having resources available to recruit a research assistant in each site who can document and facilitate the recruitment process in collaboration with the staff to ensure it is managed effectively. The sites were encouraged to contact the research team if they had any questions or if problems occurred. A research team member helped the sites complete the registrations in a couple of situations.

#### Feasibility and acceptability of the data collection process

The introduction to the tests and the collaborative discussion on ensuring reliability in the data collection were considered satisfactory. The data collectors agreed unanimously that the data collection was best done with pen and paper. It provided a convenient way of introducing the tests for the participants, and it was thought that participants would have found it challenging to answer on a tablet or PC. One DC reported:

Well… in the relation (to the participant) and being able to read their signals and for them to read our signals clearly... I think it (tablet or pc) would have been a disturbance… (DC3).

The other DCs supported this statement spontaneously.

Because we realised the importance of the paper... that they needed to see and touch it…and we talked about it. I also needed it as a “prop” to collect my thoughts.

This approach could be used in similar studies as it prompts and ensures focus and attention on the assessment for participants and DCs.

The DCs reported that carrying out the tests was relatively easy. However, the DCs could have been more open about interpreting the response to specific questions. They agreed on a strategy to write down the answer and then discuss where to “put the mark” with the other DCs to ensure it was done according to the test guidelines. For example, on the MMSE, a participant was asked which floor they were on, and the participant answered, “first floor”. It was the ground floor. However, the building was split level and raised from the ground, giving the appearance of being on the first floor. In this case, a point was given. Whether this “consensus” process is an advantage is uncertain but might indicate that new DCs must pilot-test their understanding of the tests before data collection commences. The tests were piloted, however, not with people with dementia, which might have revealed some of these issues earlier. The DCs discussed whether points should be given, which happened on only four occasions.

The data collection process was organised according to the DCs’ work schedules. For future studies, it would be preferable for DCs to collect pre- and post-data from the same individuals and sites to ensure consistency, but in this study, it was not always possible:

We could have avoided this (…that different DCs collected pre- and post-data) if there had been a planned period where resources were available for the three of us to handle things because the (data collecting) was competing with other work-related tasks. (DC1)

The collection of demographic characteristics proved challenging. It was suggested by one member of the research team (RT) that it should have been easy to fill out pre-recorded forms, and DC1 noted the following:

It is not that simple…some of these sites do not have a tradition for registering anything… it is like “come when you feel like it” … They (the sites) are asked to do something new… they will find it difficult…or think they don’t have the resources because it is an extra thing to do (DC1)

The collection of participant characteristics needs to be addressed in a more structured and with more emphasis on reliability and validity, for example through a designated staff member supporting the registration of this data. A structured recruitment process and collection of demographic data require appropriate resources and funding. However, staff at the sites were very supportive and engaged in the study.

The DCs were asked how long it took to undertake the tests. The DCs could not give an estimate but said it varied between participants. Overall, it was manageable for the participants; perhaps because the participants mostly forgot it was a test situation and acted more like they were having a conversation. Yet, according to the DCs, five tests were perceived as a maximum for one session. The DCs felt that the choice of outcomes was relevant in evaluating a school setting for people with dementia. However, they also thought it would have been pertinent to supplement the chosen outcomes by testing communication and reading skills.

I missed some scales that showed if they were better or had maintained communication level, their reading skills, that sort of things – to evaluate a school course… however, that was not what people with dementia saw as important. They focused on being social, trusting oneself, and living a life with dementia (DC1)

#### Feasibility and acceptability of outcome measures

The QOL-AD and MMSE are validated to be used with people with dementia, yet DCs did not think these tests were any more straightforward or complex than the other three tests used in this study, and none of the five caused an unacceptable level of stress. The DCs found the outcome measures to be both feasible and acceptable. Still, they expressed that the process of undertaking the measures was more challenging and ethically nuanced than anticipated.

We experienced that it does require thought not to exhaust participants or give them a negative experience, so for us… we were challenged on the relational aspect (DC1) and was seconded:

…they (the participants) needed not to feel they were being interrogated or felt tested in whether they were “good” or “bad” (DC2).

The DCs and participants found the scoring system of the measures could result in lower scores where they were not able to account for other physical or health conditions that could influence the way a participant answered; for example, one participant had challenges answering questions because of his hearing loss, rather than due to his dementia. The impact caused by average age-related cognitive decline and the impact of dementia-related cognitive deficit should be considered, as this may confound results. As well as health issues confounding potential scores, participant’s everyday skills were also identified as having a possible impact, as one DC reflects on the MMSE:

If you have been a math teacher or just good with numbers, you may ace that question where you count backwards, or if you have dyslexia, you can’t write a sentence. I mean, other tests would be better…. (DC3)

The Rosenberg Self-Esteem Scale also posed a challenge with the questions: “I feel I do not have much to be proud of”, and “At times, I think I am no good at all”. In the Danish version of the test, these questions were perceived as double negatives (with subsequent confusion), although the translation is grammatically correct. DCs found that participants could be supported to answer these questions by first establishing what a “positive” answer was and then what a “negative” answer was:

We often needed to break down, for instance, four possible questions into two categories; positive and negative – and then break it down further. (DC1).

An essential part of the process, DCs reported, was planning sufficient time to undertake the tests to ensure an ethical process was followed. The DCs found that participants needed time to understand some questions and for DCs to support the facilitation of this understanding. For example, to provide the alternative wording, the measures suggest, or as already noted, to support a dementia-friendly approach to identify “positive” or “negative” responses.

Questions on the QOL-AD, Friendship scale, Rosenberg’s self-esteem scale, and GSE scale had the unforeseen effect of prompting people with dementia to share elements of their life stories. It was also noted that these tests could provide the foundation for meaningful conversations with people with dementia. DCs suggested that the MMSE's structure and level of instruction (with its focus on correct answers rather than experiences) could be why conversation was not encouraged. The MMSE test was perceived as the most emotionally uncomfortable test for both people with dementia and the DCs.

They knew it had something to do with the disease. They had tried it a thousand times before and may not have remembered when they did it the last time, but they remembered this was not a nice feeling. Here I (the participant) felt like… here I am being confronted – and I (the DC) did not like it either. (DC2)

The DCs agreed that very few participants were excluded due to emotional toll. Emotional responses identified during the data collection related to participants’ frustrations with their declining abilities and skills. Some participants even expressed gratitude and appreciation for the conversations that touched upon memories and allowed for discussions about their past and present lives.

## Discussion

Dementia is inevitably a progressive illness. Psychosocial interventions, such as LL, which aim to support people with dementia, delay dementia’s progression, and provide meaningful activities, might be part of the answer to helping people live as well as possible with their diagnosis. Research is crucial in understanding the benefits of these interventions. This paper highlights the implications of different study designs and implementation decisions. It also explores how these can support future study design choices, particularly in examining the feasibility of a more extensive study of the LL approach for people with dementia, to inform sample sizes and optimise choices of effect measures that are reflective of and suitable for people with dementia.

### Feasibility and acceptability of the study design

The pragmatic choice of evaluating existing LL services introduced its challenges. Firstly, randomisation is often considered the best foundation for effect evaluation. However, this was impossible within the existing frames and organisation of the services. Participants had spent variable amounts of time in the services before participating in the study, possibly affecting the outcomes. Participants were also not matched. While the authors recognise that a future study would benefit from ensuring randomisation and comparable times in the service, this study also highlights the challenges of working pragmatically with existing services, where study design, meeting the needs of the service, and people with dementia need to be balanced. Such pragmatics are a part of conducting research in clinical or community settings and form part of a continuum of research from explanatory to pragmatic [43]. As randomised controlled studies require economic resources and rigorous preparatory work, this study established criteria to make an RCT possible and help define the intervention reliably.

The current results underline the need to understand not only the cause and effect but also the influences of how and where the intervention is implemented. People with dementia and their carers show great interest in participating in research about dementia. What is becoming increasingly important is that they give input into what is being researched. Holtrop and Glasgow discuss that research that falls within the pragmatic paradigm of research should consider exploring “what works in a typical clinical care setting” and ensuring that the research questions are focused on issues that patients want to explore [43]. This paper presents one way this focus can be achieved, as it drew from previous qualitative research on LL and includes a consultation process with those attending and delivering the service.

### Feasibility and acceptability of the data collection process

A review of the data collection process identified areas for future consideration. Among the participants, only three (4.4%) could not complete the tests, affirming the appropriateness of using a limited number of measures (five in this study) to avoid overburdening the participants. Using paper and pen to complete the measures also provided a focus point and was perceived to aid the process.

However, improvements are needed in documenting participant demographic information. Firstly, three participants had MCI and not dementia. This raises the question of potential misdiagnosis and how/what diagnostic information is shared/collected by services. Missing demographic data, e.g. dementia type, level of education, and engagement with other services, was also identified. This indicates the different approaches to documenting patient characteristics across the various services and municipalities. While entry criteria were discussed at the project meetings, additional staff training on recruitment or funding may have mitigated this. To further improve the completeness and validity of collecting participants’ background information, consideration could be given to involving carers, general practitioners, and/or secondary care in this process. Furthermore, the potential to include project costs for staff time may also support this process in enabling staff to dedicate hours to the task.

### Feasibility and acceptability of outcome measures

Key findings emphasised the importance of using measures informed by qualitative input from people with dementia. However, researchers chose concrete, individual tests for the constructs described by people with dementia. The choices by the researchers also involved using tests that were not explicitly validated for people with dementia. Still, they were used with people later in life, e.g. the GSE scale, Rosenberg’s self-esteem scale, and the Friendship scale. While some challenges were experienced with the phrasing of reverse questions [13], there is a precedent set by other studies for using non-validated tests for use with people with dementia, as exemplified in a multicentre randomised controlled trial that used the GSE scale as a secondary measure for evaluating a cognitive rehabilitation programme for people with early-stage dementia [44] and a study of the relationship between self-efficacy and depression with people with dementia [45, 46].

A review of measures was undertaken, and those used in this study were identified as the most appropriate at the time, given the requirements to meet the criteria set by those with dementia and the need for validated Danish translations. Since conducting this research, new measures have emerged that address some of these areas, for example the Engagement and Independence in Dementia Questionnaire [47] and the Positive Psychology Outcome Measure [48] — these offer ways to explore hope, resilience, and social engagement and are validated for use with people with dementia. The offer of a broader range of validated tests is encouraging, although many remain as English versions, so language and cultural validation remain challenges [13].

All participants completed the MMSE test, but considering the comments by the DCs, it may be worth considering using a cognitive test that does not convey the same feeling of “passing or failing” for people with dementia [13]. This poses an exciting challenge as the MMSE is one of the most widely used cognitive assessments in dementia research [45]. The MMSE has been found reliable in showing a decline. It is a test that can be administered by clinical staff quickly and has clear guidance for its use [25]. However, other studies have shown that people with dementia can experience some emotional distress when completing the MMSE [49], and that sensitivity issues apply to the outcomes, which may stem from the shift in its use as a screening test to a research tool [45]. Decisions on which measure would be most appropriate is a challenge for researchers and needs to be weighed regarding the intended use, sensitivity, time to complete, and the qualifications required to deliver each measure.

The QOL-AD test has been validated in several countries and appears valid and reliable [45]. This test, and the GSE scale’s feasibility and acceptability, was demonstrated with no missing data and a general acceptance by the DCs, who identified these tests’ ability to spark a meaningful conversation about everyday life with the participants. However, there were challenges in using measures that had not been validated for use with people with dementia, particularly regarding the phrasing of reverse questions. These were reported to cause potential confusion for the participants, thus requiring time, empathy, and explanations of the measures. The Rosenberg Self-Esteem Scale proved to be both a positive and challenging test. It opened some emotional yet meaningful conversations about life with dementia, but it also challenged understanding as the questions alternated between positive and negative statements related to self-esteem. The Friendship scale was more straightforward. All questions appeared to be understandable to the participants and brought thoughts and reflections forward about their social life when living with dementia.

### Limitations

The study was developed pragmatically to facilitate the inclusion of existing groups. This raised several challenges, such as the inability to randomise or rigorously match the control and intervention groups. This limitation may have influenced the study findings, particularly the observed higher MMSE scores in the intervention group, suggesting a potential bias toward individuals with a higher cognitive functioning level participating in the LL intervention. The lack of randomisation raises questions about the study’s internal validity and the reliability of the outcome measures in accurately assessing the effects of the LL intervention. Researchers should acknowledge the potential for confounding factors and exercise caution in attributing changes solely to the intervention, highlighting the need for a randomised design to enhance the reliability and validity of future studies. Some of the measures had not been validated for use with people with dementia; this raises concerns about what these measures truly captured and assessed. However, the measures changed little over time, showing reliability, assuming the participants’ level of functioning was relatively stable. Further limitations include challenges in capturing participant demographics and service engagement information due to constraints faced by staff. Balancing research requirements with routine activities was difficult, and future research could benefit from additional training and financial support for a dedicated liaison between services and the research team. There was a noticeable loss in follow-up data collection, with seven participants in the LL group and 11 participants in the control group having stopped participating by the final assessment (Fig. 2). These losses were not associated with the intervention itself. Given the nature of our study population, individuals with mild to moderate dementia, fluctuations in participation, and engagement are expected. The cognitive challenges faced by the participants may have contributed to the attrition, emphasising the need for flexibility and understanding in longitudinal research involving individuals with dementia.

### Implications

Our study, centred on the LL intervention, has the potential to uncover nuanced impacts on cognitive decline, emotional well-being, and overall quality of life for individuals with mild to moderate dementia. By exploring these multifaceted effects, our research contributes to a deeper understanding of how psychosocial interventions can address various aspects of participants’ lives, acknowledging the inherent variability in their responses. The findings from our feasibility study emphasise the significance of recognising individual differences in responses to interventions. Variability estimates play a pivotal role in this context, providing insights into the potential range of participant responses. By exploring factors influencing participant responses, our study contributes insights that inform the tailoring of interventions to suit individual needs better, thus enhancing overall effectiveness and participant satisfaction. Given the chronic nature of dementia, our study’s focus extends beyond short-term outcomes. By incorporating variability estimates, we aim to understand the diverse trajectories of responses over time. This approach is crucial for assessing the robustness of the effects of the LL intervention. By exploring the sustainability of the LL intervention over an extended period, our research contributes valuable insights into the potential long-term benefits and challenges associated with psychosocial interventions. This consideration is pivotal for shaping recommendations regarding integrating such programmes into routine dementia care, ensuring that our findings account for the inherent variability in individual responses and providing a foundation for personalised and effective long-term interventions. This research has the profound implication that it is feasible and imperative to recognise the valuable insights of individuals with dementia. By demonstrating that people with dementia can actively participate and provide meaningful contributions, this study emphasises the importance of incorporating their voices in shaping and determining crucial research outcomes. This recognition not only empowers individuals with dementia but also enriches the research process by ensuring a more inclusive and comprehensive understanding of the subject matter.

## Conclusion

Our research reveals nuanced impacts on cognitive decline, emotional well-being, and overall quality of life, emphasising the crucial need for tailored interventions over standardised approaches. Unlike one-size-fits-all methods, our study prioritises customisation, recognising and accommodating diverse participant responses. This insight significantly contributes to understanding long-term benefits, challenges, and the imperative for sensitive, personalised approaches in psychosocial interventions for dementia care.

Moreover, our study addresses challenges in identifying culturally and linguistically appropriate measures, stressing the importance of developing and validating inclusive tools. This not only aids in providing variability estimates for outcome measures but also ensures that findings are applicable across diverse populations, aligning with the aim of our pilot study.

### Supplementary Information

**Additional file 1.** Appendix 1 Interview guide for Data Collectors focus group

## Abbreviations

CT: Cognitive therapy
CST: Cognitive stimulation therapy
CS: Cognitive stimulation
LL: Lifelong learning
MCI: Mild cognitive impairment
DC: Data collectors
MMSE: Mini-mental state exam
QoL-AD: Quality-of-Life Alzheimer’s Disease
GSE: General Self-Efficacy Scale
