# Supplementary material for: A feasibility and pilot study of a “lifelong learning” intervention for people with dementia
Source: Pilot Feasibility Stud. 2024 May 1;10:69. doi: 10.1186/s40814-024-01493-5 (PMC11061898; doi:10.1186/s40814-024-01493-5)
Supplement: Supplementary file 1 — Additional file 1. Appendix 1 Interview guide for Data Collectors focus group [file 40814_2024_1493_MOESM1_ESM.docx]

Appendix 1 Interview guide for Data Collectors focus group

**Topic 1**

Recruitment of people with dementia

- How did you experience the data collection?

- tell me more about that.

- How do you think the administration of the data collection went?

- what did you think worked well

- what do you think should be changed

- What do you think of the engagement of the staff doing the recruitment?
- Do you think that was affected by their thoughts about the intervention?
- In this study it was staff at the services who recruited – what do you find to be advantages and what is disadvantages?

**Topic 2**

Feasibility and acceptability of the datacollection

- How do you think the data collection went?
- Do you think participants were engaged and interested in participating in the tests?
- Now, with more experience – do you think you would obtain different results with the participants?
- Which difficulties did you encounter during the collection of data? And why?
- What were the responses from participants?
- Should you have had more supervision?
- What sense do you have of how feasible it would be to carry on with a larger study?

**Topic 3**

Feasibility and acceptability of outcome measures

- What do you think of the individual tests?
- Which responses did you get from the participants on the tests?
- What did you feel administering the tests?
- Did the participants understand which construct was being addressed in each test
- Do you think these test were the right to answer the research question or were there aspects of the intervention you think also should have been covered?
- The amount of time it took to administer the tests; was that appropriate or was it too much for the participants?
